# Supplementary material for: First successful allogeneic hematopoietic stem cell transplantation in STING-associated vasculopathy of infancy—A case report
Source: Mol Ther Adv. 2026 Feb 16;34(1):201699. doi: 10.1016/j.omta.2026.201699 (PMC13148905; doi:10.1016/j.omta.2026.201699)
Supplement: Document S1. Figure S1 and Table S1 [file mmc1.pdf]

OMTA, Volume *34*

Supplemental information

First successful allogeneic hematopoietic stem cell transplantation in STING-associated vasculopathy of infancy—A case report

Uet Yu, Aiyun Song, Changying Luo, Chengjuan Luo, Xia Qin, Xiaohang Huang, Xinan Wang, Yuchen Lin, Chen Zhou, Manpin Zhang, Jing Chen, and Xiaodong Wang

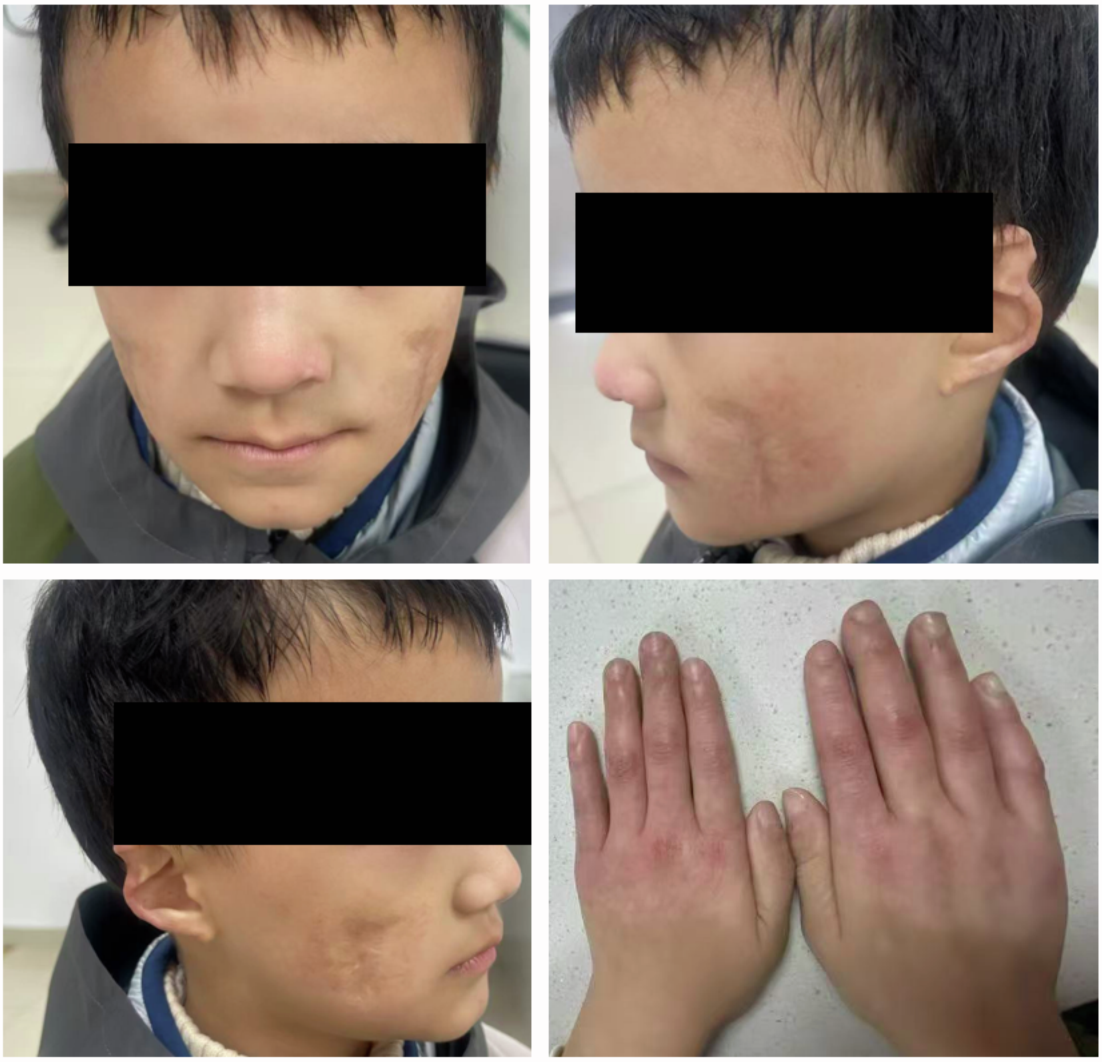

Figure S1. Cutaneous lesions 12 months after HSCT.

Clinical photos 12 months post-HSCT showing sustained resolution of the previous skin ulcerations.

Table S1. Pulmonary function of the patient before and after HSCT.

	FEV1%	FEV1/FVC%
Pre-HSCT	70.1	84.92
+ 1 month	96.80%	84.66
+ 3 months	113.3%	90.09%
+ 6 months	88.5%	100.4%
+12 months	102.4%	105.9%
