# Supplementary material for: First successful allogeneic hematopoietic stem cell transplantation in STING-associated vasculopathy of infancy—A case report
Source: Mol Ther Adv. 2026 Feb 16;34(1):201699. doi: 10.1016/j.omta.2026.201699 (PMC13148905; doi:10.1016/j.omta.2026.201699)
Supplement: Document S2. Article plus supplemental information [file mmc2.pdf]

Molecular Therapy
Advances
Case report

First successful allogeneic hematopoietic stem cell transplantation in STING-associated vasculopathy of infancy—A case report

Uet Yu,[1,2,3] Aiyun Song,[1,2,3] Changying Luo,[1,2] Chengjuan Luo,[1,2] Xia Qin,[1,2] Xiaohang Huang,[1,2] Xinan Wang,[1,2] Yuchen Lin,[1,2] Chen Zhou,[1,2] Manpin Zhang,[1,2] Jing Chen,[1,2] and Xiaodong Wang[1,2]

[1]Blood and Marrow Transplantation Center, Zhangjiang Campus, Shanghai Children's Medical Center, Shanghai Jiao Tong University School of Medicine, Shanghai, China; [2]National Children's Medical Center (Shanghai), Shanghai, China

Stimulator of interferon genes (STING)-associated vasculopathy with onset in infancy (SAVI) is a monogenic autoinflammatory disorder caused by gain-of-function mutations in TMEM173, leading to constitutive STING activation and persistent type I interferon signaling. Affected children develop early-onset cutaneous vasculopathy, systemic inflammation, and progressive interstitial lung disease, often refractory to standard immunosuppressive treatments. Janus kinase (JAK) inhibitors offer partial transient control, with risk of irreversible organ damage. Allogeneic hematopoietic stem cell transplantation (allo-HSCT) could cure SAVI by replacing the mutant immune system, but experience is very limited. We report a 6-year-old boy with SAVI carrying the TMEM173 p.N154S mutation who failed multiple JAK inhibitors, including ruxolitinib, tofacitinib, and baricitinib and developed worsening lung fibrosis and cutaneous ulcers. He underwent allo-HSCT from a fully HLA-matched sibling after myeloablative conditioning. Engraftment was rapid. By 12 months post hematopoietic stem cell transplantation (HSCT), vasculitic skin lesions had healed, lung disease was stable, and inflammatory markers normalized, and cellular and humoral immunity reconstituted, including recovery of CD8$^+$ central memory T cells and IgG and IgA. No graft-versus-host disease or major infections were observed. A transient autoimmune hemolytic anemia resolved with corticosteroids. This first successful SAVI allo-HSCT suggests curative potential via a durable immune reconstitution approach in selected patients with severe, treatment-refractory SAVI.

INTRODUCTION

Stimulator of interferon genes (STING)-associated vasculopathy with onset in infancy (SAVI) is a rare autosomal-dominant interferonopathy caused by gain-of-function variants in TMEM173, which encodes STING.[1] Aberrant STING activation drives persistent production of type I interferons and NF-κB-mediated inflammation, leading to neonatal-onset systemic inflammation, chilblain-like cutaneous vasculitic ulcerations, recurrent fever, failure to thrive, and progressive interstitial lung disease that can evolve into pulmonary fibrosis, which are associated with high morbidity and mortality in early childhood.[2] Standard treatments, including corticosteroids and off-label use of Janus kinase inhibitors such as ruxolitinib, tofacitinib, baricitinib, or an IFN-α/β receptor-blocking antibody anifrolumab, often provide only partial transient relief; inflammation may persist, and organ damage can accumulate despite therapy.[3–8] As a result, many SAVI patients progress to end-stage lung disease or other irreversible complications.

Allogeneic hematopoietic stem cell transplantation (allo-HSCT) has cured other monogenic immune disorders by replacing the diseased immune system, but its use in SAVI has been approached cautiously because of concerns about conditioning toxicity and the fact that STING is expressed in non-hematopoietic cells that would still harbor the pathogenic mutation post hematopoietic stem cell transplantation (HSCT). Only one prior case of a SAVI patient undergoing HSCT has been reported, and the patient did not survive.[9] Here, we present, to our knowledge, the first case of SAVI successfully treated with allo-HSCT. We describe the patient's clinical course and outcomes with emphasis on the mechanisms by which HSCT corrected the immune dysregulation. We also discuss the immunologic reconstitution observed post-HSCT and its correlation with disease stabilization, particularly in the lungs. This case provides important insights into the feasibility, efficacy, and limitations of HSCT as a definite therapy for severe, treatment-refractory SAVI. This study was conducted with approval from the Ethics Committee of Shanghai Children's Medical Center and in accordance with the Declaration of Helsinki.

Received 30 July 2025; accepted 11 February 2026;
https://doi.org/10.1016/j.omta.2026.201699.

[3]These authors contributed equally

Correspondence: Jing Chen, Blood and Marrow Transplantation Center, Zhangjiang Campus, Shanghai Children's Medical Center, Shanghai Jiao Tong University School of Medicine, Shanghai, China.
E-mail: chenjing@scmc.com.cn
Correspondence: Xiaodong Wang, Blood and Marrow Transplantation Center, Zhangjiang Campus, Shanghai Children's Medical Center, Shanghai Jiao Tong University School of Medicine, Shanghai, China.
E-mail: wangxiaodong@scmc.com.cn

Published by Elsevier Inc. on behalf of The American Society of Gene and Cell Therapy.
This is an open access article under the CC BY-NC-ND license (http://creativecommons.org/licenses/by-nc-nd/4.0/).

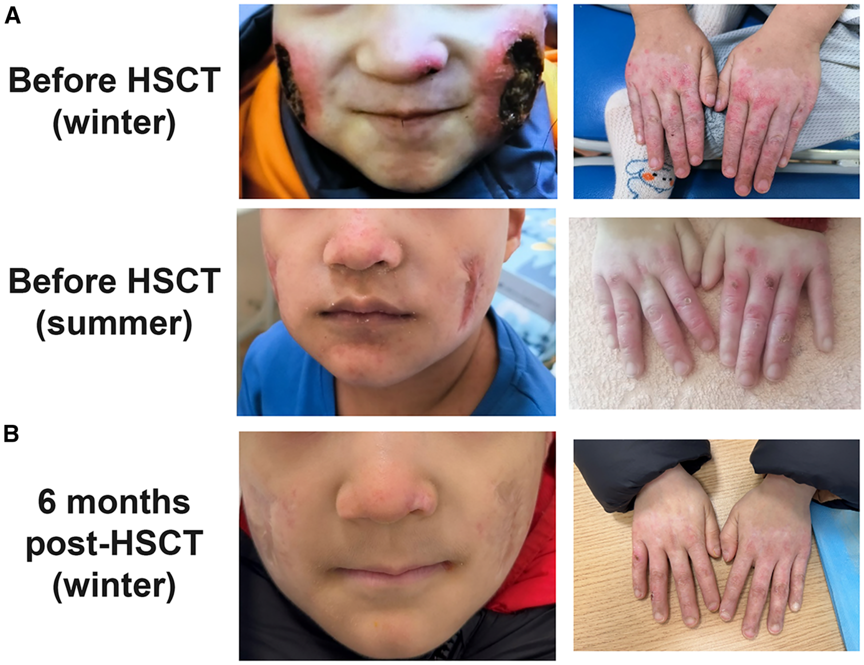

Figure 1. Cutaneous lesions before and after allogeneic HSCT
(A) Clinical photos before HSCT showing ulcerative lesions on the face and extremities. (B) Clinical photos 6 months after allogeneic HSCT, the skin ulcerations had fully re-epithelialized with only faint residual scarring.

PATIENT DETAILS AND CLINICAL FINDINGS

A 6-year-3-month-old boy presented in the neonatal period with recurrent ulcerative skin lesions on the face, buttocks, and extremities. The lesions were chilblain-like and worsened in cold weather (Figure 1A), accompanied by low-grade fevers. He also had multiple urinary tract infections in infancy. At 5 months of age, he developed fungal pneumonia (bronchoalveolar lavage positive for β-D-glucan), requiring voriconazole for 3 months. A chest computed tomography (CT) scan performed after the discontinuation of antifungal therapy showed early interstitial lung disease (ILD). At 8 months, genetic testing confirmed a heterozygous *de novo* TMEM173 missense mutation (c.461 A>G, p.N154S), establishing the diagnosis of SAVI. Inflammatory markers such as erythrocyte sedimentation rate (ESR) and IL-6 were elevated (ESR: 92.8 mm/h; IL-6: 18.98 pg/mL). Other inflammatory markers including IL2, IL-10, IL-17, IL-1β, TNF-α, IFN-α, and IFN-γ were within the normal range. ESR and IL-6 remained high even after acute fungal infection resolved, consistent with chronic autoinflammation.

By 13 months of age, treatment with the Janus kinase (JAK)1/2 inhibitor ruxolitinib (2.5 mg/kg twice daily) was initiated. This led to reduction in fever frequency, while the skin vasculitic ulcers and ILD persisted. After 17 months on ruxolitinib, the drug was stopped due to ongoing active vasculitis and hepatotoxicity (elevated ALT and AST >3× upper limit of normal). The patient was then switched to tofacitinib (5 mg daily). Over the next 28 months, his cutaneous lesions continued to cycle seasonally (worse in winter) (Figure 1A). Chest CT scans during this period demonstrated bilateral ground-glass opacities, developing panlobular emphysema, and evolving fibrosis (Figure 2). At 4 years of age, pirfenidone (an anti-fibrotic agent, 200 mg daily) was added for ILD, and another JAK1/2 inhibitor, baricitinib (1 mg daily), was introduced to augment the anti-inflammatory regimen. Despite some radiographic stabilization of the lung findings, the child's pulmonary function showed a gradual decline. The forced expiratory volume in 1 s (FEV1) fell from 82.8% to 70.1% of the predicted value. The diffusion capacity of the lungs for carbon monoxide (DLCO) was not measured due to young age. His growth also plateaued over 12 months. The mean daily diary score (DDS) for SAVI-related symptoms was 1.16 (scoring fatigue 1, respiratory difficulty 1, and ulcer severity 2 on a 0–3 scale).

Given the patient's refractory multi-organ disease and progressive pulmonary fibrosis, a comprehensive evaluation was undertaken to consider allo-HSCT as a definitive therapy. Multidisciplinary discussions were held with hematologists and immunologists at our center, Beijing Children's Hospital, and Hong Kong Children's Hospital. The consensus was that allo-HSCT may offer a chance to halt the disease course by restoring a normal immune system. After thorough counseling of the family regarding the risks and potential benefits, we proceeded with allo-HSCT to replace the patient's dysfunctional hematopoietic and immune system.

Immunophenotyping prior to HSCT revealed an immune profile consistent with chronic interferon-driven inflammation. Notably, the patient had an extremely low count of $CD8^+$ central memory T (TCM) cells (only 0.54 cells/μL, markedly below the normal range of 7.52–54.43 cells/μL), along with persistently elevated ESR (97 mm/h) and serum IL-6 levels (8.46 mmol/L) (Figures 3A, 3B, and 3F). There was also evidence of B cell and monocyte activation. Total IgG (27.5 g/L) and IgA (8.33 g/L) (Figure 3G) were elevated above age-normal ranges although autoantibody testing (ANA, c-ANCA, and p-ANCA) was negative. These findings suggested ongoing immune activation and dysregulation in both innate and adaptive compartment, despite multiple attempts of immunosuppressive therapy.

A fully HLA-matched (10/10) older sister served as a donor. Myeloablative conditioning included busulfan (4.2 mg/kg/day × 3 days),

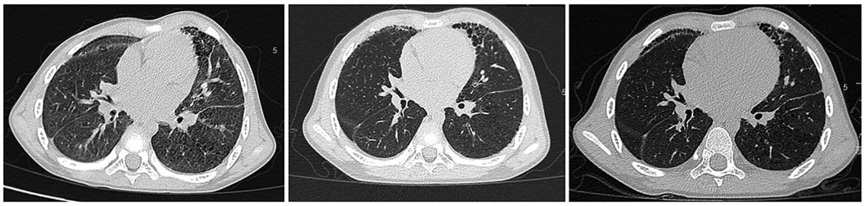

Figure 2. Serial chest CT scans before and after HSCT

High-resolution chest CT images obtained before HSCT, 3 and 9 months after HSCT, showing bilateral ground-glass opacities, panlobular emphysema, and fibrotic changes with similar extent and distribution at each time point.

cyclophosphamide (60 mg/kg/day × 2 days), fludarabine (45 mg/m^2/day × 4 days), thiotepa (10 mg/kg/day × 1 day), and anti-thymocyte globulin (ATG, 2.5 mg/kg/day × 3 days). Peripheral blood stem cells (total neutrophil count, TNC, 12.62 × 10^8/kg; $CD34^+$ cell, 12.48 × 10^6/kg) were infused, and graft-versus-host disease (GVHD) prophylaxis employed cyclosporine A (CSA, initial dost at 150 mg/day, adjusted to serum levels of 150–200 ng/mL) and methotrexate (10 mg/m^2 on days +1, +3, +6).

The regimen was well tolerated. Neutrophil engraftment occurred by day +14 and platelets by day +21. Day +30 chimerism showed >99% donor cells in total and T cell compartments, with no acute GVHD or peri-transplant infections. On day +50, the patient developed transient autoimmune hemolytic anemia (AIHA), which resolved promptly with steroid (methylprednisolone 0.6 mg/kg/day for 1 week then tapered over 1 month) without altering immunosuppression.

By 6 months post-HSCT, the chronic skin ulcers had fully healed, leaving only faint residual scars (Figure 1B), and remained resolved at 12 months post-HSCT (Figure S1). To assess whether IFN-driven inflammation had been durably suppressed after immune replacement, we serially monitored systemic inflammatory markers and cytokines. Although ESR and IL-6 showed brief fluctuations in the early post-HSCT period, both declined steadily thereafter, reaching near-normal levels by 9 months and remaining low at 12 months post-HSCT (ESR 27 mm/h; IL-6 2.0 pg/mL) (Figures 3A and 3B). Other cytokines relevant to type I IFN-mediated inflammation, including IL-2, IL-10, TNF-α, IFN-α, IFN-γ, and IL-17, were also monitored (Figure 3B). These cytokines remained within normal limits throughout follow-up, with only minor transient variation after HSCT. Taken together, the sustained normalization of inflammatory markers and cytokine profiles supports effective down-regulation of the previously overactive type I IFN pathway.

To determine whether hematopoietic replacement translates into global immune stabilization, we tracked immune reconstitution. Complete blood counts showed white blood cell counts stabilizing around 4–5 × 10^9/L from 1 month post-HSCT onward (Figure 3C). Neutrophils recovered rapidly after engraftment and remained in the normal range thereafter. Lymphocyte recovery was slower, as expected after myeloablative HSCT, but counts rose progressively and reached the lower end of normal by 12 months post-HSCT. Other innate subsets, including monocytes, eosinophils, and basophils, remained within normal ranges without HSCT (Figure 3C). These trends are consistent with early innate recovery followed by gradual adaptive reconstitution and match the patient's steady clinical improvement.

A more detailed immunophenotyping of lymphocytes was monitored post-HSCT (Figures 3D, 3E, and 3F). Specifically, we measured naive and memory $CD4^+$ and $CD8^+$ T cell subsets by flow cytometry before and after HSCT. Total $CD4^+$ counts rose gradually after HSCT. Naive $CD4^+$ cells fell early and then rebounded from ∼3 months and continued to increase. $CD4^+$ TCM cells and effector $CD4^+$ T cells showed a brief dip at 2–3 months and then steady recovery and a plateau from ∼6 months (Figure 3E). For $CD8^+$ cells, total counts rose slowly and reached the normal range by 12 months. Both naive and effector $CD8^+$ cells fell at 1 month after conditioning. Naive $CD8^+$ cells stayed low through 6 months and increased by 12 months but remained just below normal. $CD8^+$ TCM fluctuated within the normal range. Effector $CD8^+$ T cells rebounded early and expanded steadily, reaching about twice the upper limit by 12 months (Figure 3F). These patterns suggest expected early contraction from conditioning, rapid effector expansion with reduced inflammation, and delayed thymic output of naive cells.

Both IgG and IgA decreased to 11.4 and 2.34 g/L, respectively, within normal ranges by 12 months post-HSCT (Figure 3G). We also measured B cells and plasma cells before and after HSCT (Figure 3D). B cells rose slowly and reached the normal range by ∼6 months and then stayed stable. Plasma cells showed a brief peak at ∼1 month and returned to the normal range by the 3-month visit, remaining stable thereafter. IgE levels were remained within normal range (<200 IU/L) before and after HSCT (Figure 3H). These data support gradual humoral and cellular reconstitution after HSCT.

At his last follow-up (12 months post-HSCT), he remained clinically stable and demonstrated catch-up growth, and chest CT showed unchanged fibrosis with no new interstitial lesions (Figure 2). Pulmonary function remained stable and improved over time, increasing to 102.40% predicted at 12 months for FEV1 (Table S1). His mean DDS score decreased to 0.16, reflecting only mild intermittent respiratory symptoms and complete resolution of skin disease. Functionally, he can now walk independently and run short distances, which was not possible before HSCT. CSA tapering was initiated at 8 months post-HSCT, and the CSA trough level was reduced to approximately 40–50 ng/mL.

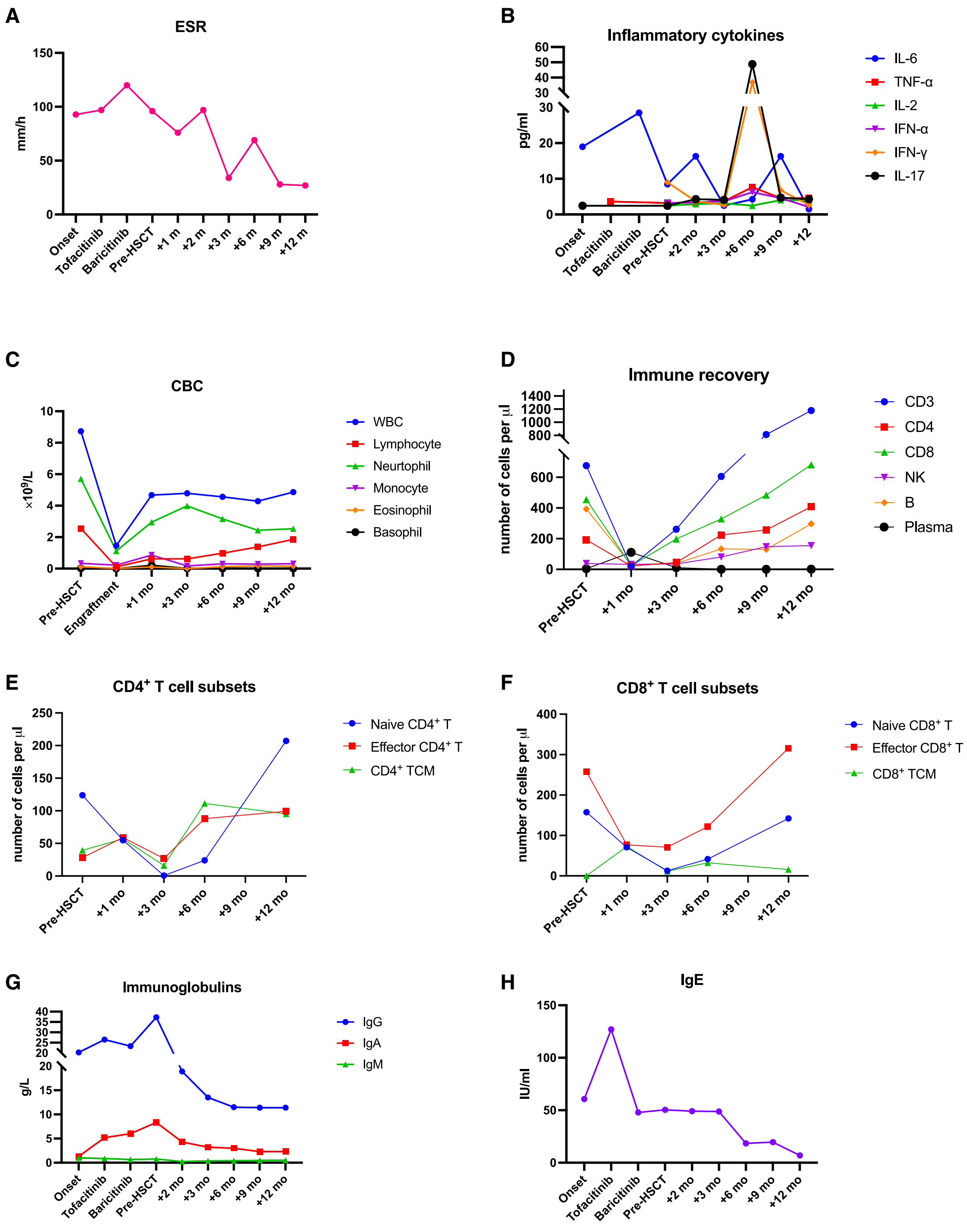

Figure 3. Longitudinal inflammatory markers and cellular and humoral parameters before and after allogeneic HSCT

(A) ESR and (B) serum inflammatory cytokines (IL-6, TNF-α, IL-2, IFN-α, IFN-γ, and IL-17) measured from disease onset, during tofacitinib and baricitinib treatment, immediately before HSCT (pre-HSCT), and at 1, 3, 6, 9, and 12 months post-HSCT (normal reference ranges are ESR 0–20 mm/h, IL-6 <5.4 pg/mL, TNF-α <8.0 pg/mL, IL-2 <7.5 pg/mL, IFN-α <20 pg/mL, IFN-γ <23.1 pg/mL, and IL-17 <21.4 pg/mL). (C) Complete blood counts (white blood cell counts (WBCs), lymphocytes, neutrophils, monocytes, eosinophils, and basophils) pre-HSCT, at engraftment, and at 1, 3, 6, 9, and 12 months post-HSCT (normal reference ranges are WBCs 4.3–11.3 × 10^9/L, lymphocytes 1.5–4.6 × 10^9/L, neutrophils 1.6–7.8 × 10^9/L, monocytes 0.13–0.76 × 10^9/L, eosinophils 0.00–0.68 × 10^9/L, and basophils 0.00–0.07 × 10^9/L). (D–F) Absolute counts of (D) major lymphocyte populations (CD3$^+$ T cells, CD4$^+$ T cells, CD8$^+$ T cells, natural killer [NK] cells, CD19$^+$ B cells, and plasma cells) (normal reference

(legend continued on next page)

DISCUSSION

This case describes the first successful use of allo-HSCT in a child with SAVI, demonstrating that HSCT can effectively extinguish the aberrant interferon-driven inflammation underlying this disease. Following myeloablative conditioning and engraftment of a healthy donor hematopoietic and immune system, our patient experienced complete resolution of cutaneous vasculitis, normalization of inflammatory biomarkers, and arrest of further lung fibrosis progression. These outcomes are especially remarkable given his severe pre-transplant condition—advanced ILD and ongoing skin ulceration despite prolonged therapy with multiple JAK inhibitors. Our findings provide a proof of concept that replacing the patient's dysregulated immune cells can "reset" the immune system and induce a durable remission in SAVI.

It is important to interpret this result in the context of prior experiences and the underlying disease biology. SAVI is driven by gain-of-function mutations in T*MEM173* that lock STING into a constitutively active conformation, leading to relentless production of type I interferons and NF-κB-mediated inflammation.[1,10–13] STING is highly expressed not only in immune cells but also in non-hematopoietic cells, such as endothelial cells lining blood vessels and lung epithelial and endothelial cells, where mutant STING amplifies local cytokine release and adhesion-molecule upregulation, perpetuating endothelial injury and tissue fibrosis.[14] This widespread expression initially raised concerns that HSCT might not be curative, because mutant STING in non-hematopoietic cells could continue to drive inflammation even after the bone marrow is replaced. Although directly targeting non-hematopoietic STING remains challenging, murine models carrying the p.Asn153Ser (N153S) mutation demonstrate that lung pathology is largely mediated by hematopoietic-derived T cells.[15] Consistent with this, organ-specific interventions have not cured SAVI. For example, four SAVI patients who underwent lung transplantation developed persistent interferon-mediated inflammation in their transplanted lungs, presumably due to circulating or resident immune cells still bearing mutant STING. These observations suggest that simply replacing a target organ, such as the lungs or skin, without correcting the immune system is insufficient. The inflammatory milieu will persist as long as hematopoietic cells are diseased. Conversely, replacing the source of aberrant interferon signaling, the immune system itself, is more likely to provide long-term disease control.[16,17]

Recognizing that the hyperactive immune compartment is the principal driver of SAVI, we and our colleagues at Beijing Children's Hospital and Hong Kong Children's Hospital recommended allo-HSCT to reconstitute a wild-type hematopoietic system. After in-depth discussions with the team and the family, allo-HSCT was pursued. Myeloablative conditioning and infusion of an HLA-matched sibling stem cells achieved full donor chimerism, resolution of systemic interferon markers, and complete healing of vasculitic lesions, despite the ongoing presence of mutant STING in non-hematopoietic tissues. Our ongoing observations support the concept that "resetting" the immune system can halt systemic and pulmonary inflammation in SAVI. Longer follow-up will determine whether this approach also stabilizes or reverses organ-specific, tissue-resident disease manifestations.

These findings underscore that replacing the patient's dysregulated immune system is key to halt SAVI. Standard treatments—corticosteroids and JAK inhibitors—often provide only partial, transient relief and fail to prevent irreversible organ injury.[5] Recently, single-cell RNA sequencing of peripheral blood mononuclear cells (PBMCs) from SAVI patients identified a distinct group of "disease-associated" monocytes with strong activation of type I IFN and NF-κB pathways and high production of IL-6 and CCL3/CCL4. These monocytes chronically stimulate T cells, leading to their exhaustion and death. Their STING-driven signature is only partly dependent on type I IFN, suggesting that JAK/IFN-targeted drugs alone may not fully correct the abnormal hematopoietic cells.[18]

Our patient's improvement after HSCT confirms that the hematopoietic compartment is a principal driver of SAVI pathology and that replacing this compartment can "reset" systemic inflammation even when non-hematopoietic cells still express mutant STING. Complementary mouse work using conditioning STING gain-of-function models shows that endothelial STING expression is sufficient to initiate lung lymphocytic infiltration and chemokine production, but the full lung phenotype depends on recruitment and activation of inflammatory monocytes and T cells.[19] Furthermore, one recent case with novel F269S STING variant demonstrates spontaneous upregulation of interferon and inflammatory pathways in bone marrow cells and T cell compartment, while mutant immune cells secrete cytokines that fuel endothelial activation and damage, highlighting the hematopoietic system as a continuous source of injury signals.[20]

Genotype-phenotype correlations in SAVI suggest that certain variants (e.g., p.N154S, p.V155M, and R281Q) are associated with a more aggressive course.[21–23] In such patients, early consideration of HSCT may be warranted to forestall end-stage fibrosis.

ranges are $CD3^+$ T cells 883.59–2,478.77 cells/μL, $CD4^+$ T cells 432.41–1,402.16 cells/μL, $CD8^+$ T cells 281.49–818.13 cells/μL, NK cells 74.03–366.37 cells/μL, B cells 121.00–347.43 cells/μL, and plasma cells 0.10–7.01). (E) $CD4^+$ T cell subsets (naive $CD4^+$ T cells, effector $CD4^+$ T cells, and $CD4^+$ central memory [TCM] cells) and (F) $CD8^+$ T cell subsets (naive $CD8^+$ T cells, effector $CD8^+$ T cells, and $CD8^+$ TCM cells) pre-HSCT and at 1, 3, 6, 9, and 12 months post-HSCT (normal reference ranges: naive $CD4^+$ T cells 283.34–1,131.97 cells/μL, effector $CD4^+$ T cells 16.63–53.12 cells/μL, and $CD4^+$ TCM cells 96.22–223.55 cells/μL; naive $CD8^+$ T cells 193.29–525.93 cells/μL, effector $CD8^+$ T cells 24.60–166.89 cells/μL, and $CD8^+$ TCM cells 2.11–11.17 cells/μL). (G and H) Serum immunoglobin levels (IgG, IgA, IgM, and IgE) from onset of disease through JAK inhibitor treatment, pre-HSCT, and at 1, 3, 6, 9, and 12 months post-HSCT (normal reference ranges: IgG 6.7–15.3 g/L, IgA 0.52–2.74 g/L, IgM 0.48–2.31 g/L, and IgE <200 IU/mL).

By contrast, those with milder mutations, such as p.C147Y, C617A, and R284T, may be managed longer with medical therapy, reserving transplant for disease progression.[8,21,24,25] Nevertheless, once clinical indicators of irreversible damage, such as declining pulmonary function or multiorgan failure, appear, HSCT should be discussed as a potentially curative intervention.[12,26]

An important challenge is distinguishing true remission from the effects of peri-transplant immunosuppression. However, the child's condition continued to improve while immunosuppressants were tapered, strongly suggesting that the remission is maintained by the new immune system itself rather by pharmacologic suppression. Supporting this, we documented >99% donor chimerism in peripheral blood and a sustained normalization of interferon-driven markers, even during the period when the patient experienced AIHA that required transient steroids. We also saw normalization of B cells and immunoglobulin levels and reconstitution of T cell memory populations within 6–12 months post-HSCT, indicating robust restoration of both humoral and cellular immunity.

Although established lung fibrosis cannot be fully reversed, stabilization of pulmonary function represents a critical therapeutic milestone.[14,26,27] In our patient, FEV1, which had been declining before HSCT, showed a gradual recovery and reached 102.4% of predicted value by 12 months post-HSCT, effectively halting the pre-transplant decline. Notably, DLCO measurement was not performed before and after HSCT, as the patient was too young to reliably corporate with testing. Nonetheless, our observations suggest that earlier transplantation—before advanced fibrosis develops—may offer even greater respiratory benefit. The main limitation of our report is the relatively short follow-up, currently only 1 year after HSCT. Long-term follow-up will clarify whether incremental lung improvement in pulmonary function is possible and will allow careful monitoring for any late complications.

Beyond SAVI, this experience holds promise for other interferonopathies and autoinflammatory syndromes that defy medical therapy. HSCT may offer a one-time curative strategy for disorders driven by dysregulated innate immunity, including coatoma subunit α (COPA) syndrome, chronic atypical neutrophilic dermatosis with lipodystrophy and increased temperature (CANDLE) syndrome, or familial chilblain lupus.[28] Moving forward, collaborative registries and prospective studies will be crucial to refine transplant conditions, conditioning regimens, and long-term management, while emerging approaches such as gene therapy may provide future alternatives.

In summary, allo-HSCT cured a child with severe, treatment-refractory SAVI by fully replacing his defective immune system and restoring interferon balance. This patient achieved lasting remission, healed vasculopathy, and halted lung fibrosis after failing multiple JAK inhibitors. These results underscore the value of early genetic screening to identify high-risk SAVI mutations and support timely HSCT referral—offering a potential cure where medical therapy alone falls short.

ACKNOWLEDGMENTS

We sincerely thank Dr. Pamela Pui-Wah Lee from Hong Kong Children's Hospital and Dr. Huawei Mao from Peking Children's Hospital for their invaluable contributions to the decision-making process and for collaboratively formulating the HSCT treatment plan for this patient. We are also deeply grateful to the patient's family for their courage, trust, and cooperation in enabling us to undertake the first HSCT procedure for a patient with SAVI.

AUTHOR CONTRIBUTIONS

U.Y. and A.S. contributed equally to this work and wrote the draft of this manuscript. X.Q., X.H., Xinan Wang, Y.L., C.Z., and M.Z. contributed to the collection of clinical data and the preparation of figures. Changying Luo and Chengjuan Luo gave critical reviews of the manuscript. J.C. and Xiaodong Wang conceived the study and revised the draft of this manuscript.

DECLARATION OF INTERESTS

The authors declare no competing interests.

SUPPLEMENTAL INFORMATION

Supplemental information can be found online at https://doi.org/10.1016/j.omta.2026.201699.

REFERENCES

1. Liu, Y., Jesus, A.A., Marrero, B., Yang, D., Ramsey, S.E., Sanchez, G.A.M., Tenbrock, K., Wittkowski, H., Jones, O.Y., Kuehn, H.S., et al. (2014). Activated STING in a Vascular and Pulmonary Syndrome. N. Engl. J. Med. *371*, 507–518. https://doi.org/10.1056/nejmoa1312625.

2. Chia, J., Eroglu, F.K., Özen, S., Orhan, D., Montealegre-Sanchez, G., de Jesus, A.A., Goldbach-Mansky, R., and Cowen, E.W. (2016). Failure to thrive, interstitial lung disease, and progressive digital necrosis with onset in infancy. J. Am. Acad. Dermatol. *74*, 186–189. https://doi.org/10.1016/j.jaad.2015.10.007.

3. Jianqiang, W., Qing, Z., Hua, Z., and Meiping, L. (2023). Case report: JAK1/2 inhibition with baricitinib in the treatment of STING-associated vasculopathy with onset in infancy. Pediatric Rheumatology Online Journal *21*, 131. https://doi.org/10.1186/s12969-023-00916-6.

4. Danhong, S., Xue-Gong, F., Qing, Z., Xuefeng, X., and Meiping, L. (2022). Use of Tofacitinib for infant-onset STING-associated vasculopathy: A case report from China. Medicine *101*, e31832. https://doi.org/10.1097/md.0000000000031832.

5. YunFan, D., YunFan, D., Xiuyun, L., Xiu-yun, L., Zhigang, Z., ZhiPeng, Z., Jianxin, H., Jian-Xin, H., Qin, Y., and Qing-Qin, Y. (2020). Stimulator of Interferon Genes-Associated Vasculopathy With Onset in Infancy: A Systematic Review of Case Reports. Front. Pediatr. *8*, 577918. https://doi.org/10.3389/fped.2020.577918.

6. Kretzschmar, G., Páez, L.P., Tan, Z., Wang, J., Gonzalez, L., Mugabo, C.H., Johnsson, A., Chen, Y., Mikeš, J., Lakshmikanth, T., et al. (2024). Normalized Interferon Signatures and Clinical Improvements by IFNAR1 Blocking Antibody (Anifrolumab) in Patients with Type I Interferonopathies. J. Clin. Immunol. *45*, 31. https://doi.org/10.1007/s10875-024-01826-2.

7. Alehashemi, S., Buehring, B., de Jesus, A.A., Gaurav, S., Rastegar, A., Baumgardner, A., Friend, K., Arisa, O.T., Figg, W.D., Fink, D., et al. (2025). Sustained Interferon Signature Suppression With Anifrolumab in a Patient With STING-Associated Vasculopathy with Onset in Infancy Refractory to JAK Inhibitor and Dazukibart Therapy. Arthritis Rheumatol. *77*, 1087–1091. https://doi.org/10.1002/art.43145.

8. Mansilla-Polo, M., Martín-Torregrosa, D., Escutia-Muñoz, B., Torres-Navarro, I., Saro-Buendía, M., and Botella-Estrada, R. (2024). Successful Treatment of Stimulator of Interferon Genes-Associated Vasculopathy of Infantile Onset SAVI Syndrome With Anifrolumab. JAMA Dermatol. *160*, 899–901. https://doi.org/10.1001/jamadermatol.2024.2160.

9. Cooray, S., Henderson, R., Solebo, A.L., Ancliffe, P., Eleftheriou, D., and Brogan, P.A. (2021). Retinal vasculopathy in STING-associated vasculitis of infancy

(SAVI). Rheumatology *60*, e351–e353. https://doi.org/10.1093/rheumatology/keab297.

10. Hannah, S., Hannah, S., Axel, R., Axel, R., Angela, R.W., Angela, R.-W., Hella, L., and Hella, L. (2020). Type I interferon-independent T cell impairment in a Tmem173 N153S/WT mouse model of STING associated vasculopathy with onset in infancy (SAVI). Clin. Immunol. *216*, 108466. https://doi.org/10.1016/j.clim.2020.108466.
11. Anne Louise, H., Anne Louise, H., Kojiro, M., Kojiro, M., Francisco, J.S., Francisco, J.S., Tomohiko, T., Tomohiko, T., Christian, K.H., and Christian, K.H. (2019). STING palmitoylation as a therapeutic target. Cell. Mol. Immunol. *16*, 236–241. https://doi.org/10.1038/s41423-019-0205-5.
12. Li, W., Wang, W., Wang, W., Zhong, L., Gou, L., Wang, C., Ma, J., Quan, M., Jian, S., Tang, X., et al. (2022). Janus Kinase Inhibitors in the Treatment of Type I Interferonopathies: A Case Series From a Single Center in China. Front. Immunol. *13*, 825367. https://doi.org/10.3389/fimmu.2022.825367.
13. Gómez-Arias, P.J., Gómez-García, F., Hernández-Parada, J., Montilla-López, A.M., Ruano, J., and Parra-Peralbo, E. (2021). Efficacy and Safety of Janus Kinase Inhibitors in Type I Interferon-Mediated Monogenic Autoinflammatory Disorders: A Scoping Review. Dermatol. Ther. *11*, 733–750. https://doi.org/10.1007/s13555-021-00517-9.
14. Clémence, D., David, C., Marie-Louise, F., and Marie-Louise, F. (2022). Lung Inflammation in STING-Associated Vasculopathy with Onset in Infancy (SAVI). Cells *11*, 318. https://doi.org/10.3390/cells11030318.
15. James, D.W., James, D.W., Ricardo, A.I.-C., Ricardo, A.I.-C., Brock, G.B., Brock, G.B., Teresa, L.A., Teresa, L.A., Teresa, L.A., Amber, M.S., et al. (2017). STING-associated vasculopathy develops independently of IRF3 in mice. J. Exp. Med. *214*, 3279–3292. https://doi.org/10.1084/jem.20171351.
16. Dohyung, K., Dohyung, K., Kun Baek, S., Kun Baek, S., Eom Ji, C., Eom Ji, C., Jinho, Y., and Jinho, Y. (2022). A 17-Year-Old Girl Diagnosed With STING-Associated Vasculopathy With Onset in Infancy (SAVI) After Lung Transplantation. Chest *162*, e249–e252. https://doi.org/10.1016/j.chest.2022.05.025.
17. Berrada, K.R., Belot, A., Neven, B., Ohlmann, C., Tronc, F., Rice, G., Thouvenin, G., Dubus, J.C., Mazenq, J., Frémond, M.L., et al. (2023). Lung Transplantation under a Janus Kinase Inhibitor in Three Patients with SAVI Syndrome. J. Clin. Immunol. *43*, 2156–2164. https://doi.org/10.1007/s10875-023-01595-4.
18. De, C.C., Delage, L., Batignes, M., Riller, Q., Luka, M., Remaury, A., Sorin, B., Fali, T., Masson, C., Hoareau, B., et al. (2023). Single-cell RNA-sequencing of PBMCs from SAVI patients reveals disease-associated monocytes with elevated integrated stress response. Cell Rep. Med. *4*, 101333. https://doi.org/10.1016/j.xcrm.2023.101333.
19. Kevin MingJie, G., Kevin, M.G., Mona, M., Mona, M., Thomas, T., Thomas, T., Ann, M.R., Ann, M.-R., Katherine, A.F., and Katherine, A.F. (2022). Radioresistant cells initiate lymphocyte-dependent lung inflammation and IFNγ-dependent mortality in STING gain-of-function mice. Proc. Natl. Acad. Sci. USA *119*, e2202327119. https://doi.org/10.1073/pnas.2202327119.
20. Valeri, E., Breggion, S., Barzaghi, F., Abou Alezz, M., Crivicich, G., Pagani, I., Forneris, F., Sartirana, C., Costantini, M., Costi, S., et al. (2024). A novel STING variant triggers endothelial toxicity and SAVI disease. J. Exp. Med. *221*, e20232167. https://doi.org/10.1084/jem.20232167.
21. Marie-Louise, F., Marie-Louise, F., Alice, H., Alice, H., Alice, H., Laureline, B., Laureline, B., Isabelle, M., Isabelle, M., Isabelle, M., et al. (2020). Overview of STING-Associated Vasculopathy with Onset in Infancy (SAVI) Among 21 Patients. J. Allergy Clin. Immunol. Pract. *9*, 803–818.e11. https://doi.org/10.1016/j.jaip.2020.11.007.
22. Yan, W., Yan, W., Wei, F., Fan, W., Xiaolei, Z., and Xiaolei, Z. (2021). STING-associated vasculopathy with onset in infancy: a familial case series report and literature review. Ann. Transl. Med. *9*, 17. https://doi.org/10.21037/atm-20-6198.
23. Rensheng, W., Rensheng, W., Johannes, F., Johannes, F., Ia, Z., Ia, Z., Min Ae, L.-K., Min Ae, L.K., Christine, W., Christine, W., et al. (2022). Phenotypic spectrum in recessive STING-associated vasculopathy with onset in infancy: Four novel cases and analysis of previously reported cases. Front. Immunol. *13*, 1029423. https://doi.org/10.3389/fimmu.2022.1029423.
24. Latour-Álvarez, I., Murcia-Clemente, L., Vázquez, I.P., Garramone-Ramírez, J.E., Clemente, D., Sanz, V., and Torrelo, A. (2024). STING-associated vasculopathy with onset in infancy (SAVI) presenting with skin lesions. Pediatr. Dermatol. *41*, 893–896. https://doi.org/10.1111/pde.15620.
25. Barry, K.K., Kranseler, J.S., and Robinson, S.N. (2024). A case of STING-associated vasculopathy with onset in infancy with novel STING1 variant. Pediatr. Dermatol. *41*, 697–700. https://doi.org/10.1111/pde.15559.
26. Konstantinos, D., Roubini, S., Charalampos, S., and Clio, P.M. (2023). STING-Associated Vasculopathy with Onset in Infancy: A Review Focusing on Pathophysiology and Treatment Options. J. Mol. Pathol. *4*, 294–306. https://doi.org/10.3390/jmp4040024.
27. Marie-Louise, F., Marie-Louise, F., Yanick, J.C., and Yanick, J.C. (2021). STING-Mediated Lung Inflammation and Beyond. J. Clin. Immunol. *41*, 501–514. https://doi.org/10.1007/s10875-021-00974-z.
28. Goldbach-Mansky, R., Alehashemi, S., and de Jesus, A.A. (2025). Emerging concepts and treatments in autoinflammatory interferonopathies and monogenic systemic lupus erythematosus. Nat. Rev. Rheumatol. *21*, 22–45. https://doi.org/10.1038/s41584-024-01184-8.

OMTA, Volume *34*

Supplemental information

First successful allogeneic hematopoietic stem cell transplantation in STING-associated vasculopathy of infancy—A case report

Uet Yu, Aiyun Song, Changying Luo, Chengjuan Luo, Xia Qin, Xiaohang Huang, Xinan Wang, Yuchen Lin, Chen Zhou, Manpin Zhang, Jing Chen, and Xiaodong Wang

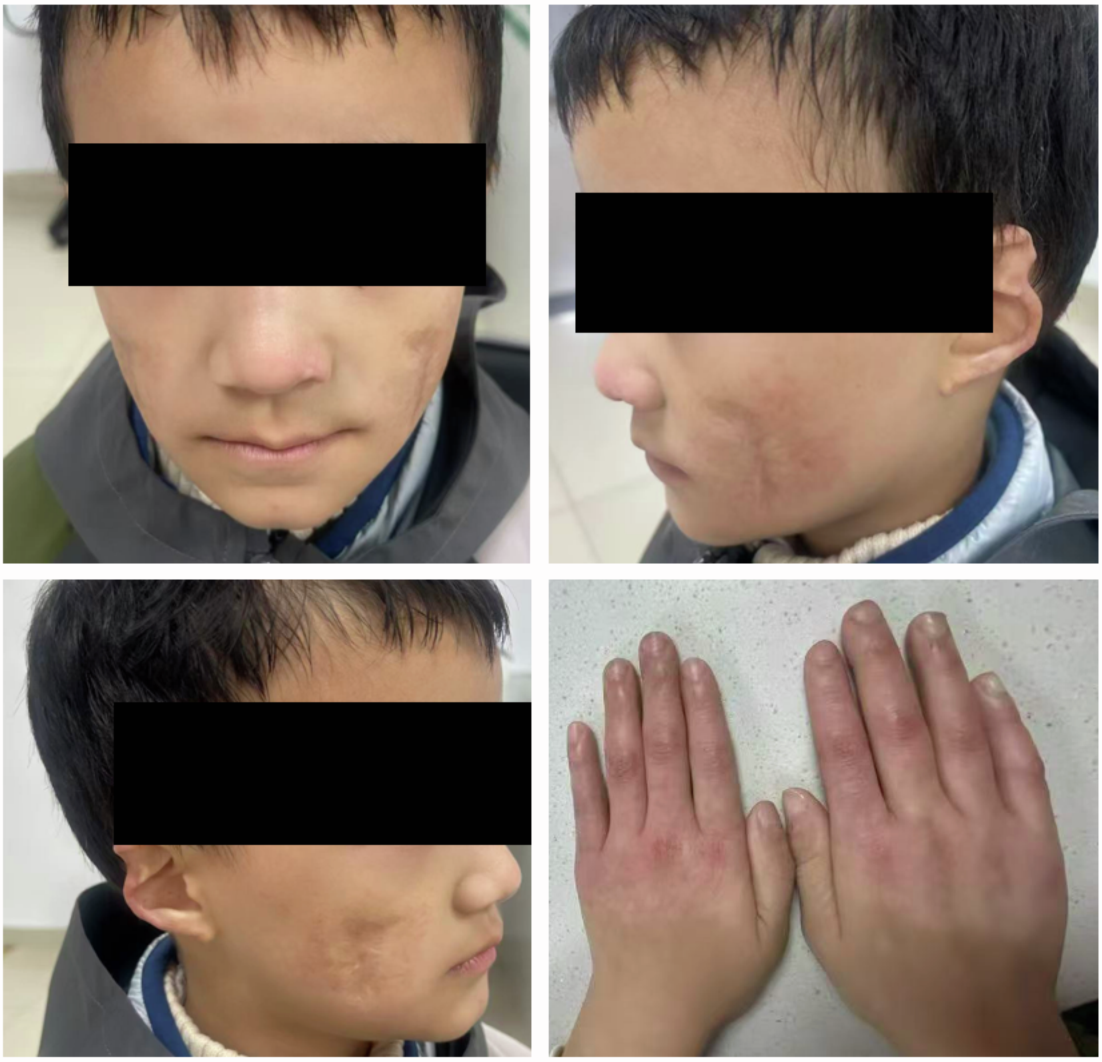

Figure S1. Cutaneous lesions 12 months after HSCT.

Clinical photos 12 months post-HSCT showing sustained resolution of the previous skin ulcerations.

Table S1. Pulmonary function of the patient before and after HSCT.

	FEV1%	FEV1/FVC%
Pre-HSCT	70.1	84.92
+ 1 month	96.80%	84.66
+ 3 months	113.3%	90.09%
+ 6 months	88.5%	100.4%
+12 months	102.4%	105.9%
